# Refractive Development in the “ROP Rat”

**DOI:** 10.1155/2012/956705

**Published:** 2012-02-08

**Authors:** Toco Y. P. Chui, David Bissig, Bruce A. Berkowitz, James D. Akula

**Affiliations:** ^1^Department of Ophthalmology, Children's Hospital Boston and Harvard Medical School, 300 Longwood Avenue, Fegan 4, Boston, MA 02115, USA; ^2^Department of Anatomy and Cell Biology, Wayne State University School of Medicine, Detroit, MI 48201, USA; ^3^Department of Ophthalmology, Wayne State University School of Medicine, Detroit, MI 48201, USA

## Abstract

Although retinopathy of prematurity (ROP) is clinically characterized by abnormal retinal vessels at the posterior pole of the eye, it is also commonly characterized by vascular abnormalities in the anterior segment, visual dysfunction which is based in retinal dysfunction, and, most commonly of all, arrested eye growth and high refractive error, particularly (and paradoxically) myopia. The oxygen-induced retinopathy rat model of ROP presents neurovascular outcomes similar to the human disease, although it is not yet known if the “ROP rat” also models the small-eyed myopia characteristic of ROP. In this study, magnetic resonance images (MRIs) of albino (Sprague-Dawley) and pigmented (Long-Evans) ROP rat eyes, and age- and strain-matched room-air-reared (RAR) controls, were examined. The positions and curvatures of the various optical media were measured and the refractive state (*℞*) of each eye estimated based on a previously published model. Even in adulthood (postnatal day 50), Sprague-Dawley and Long-Evans ROP rats were significantly myopic compared to strain-matched controls. The myopia in the Long-Evans ROP rats was more severe than in the Sprague-Dawley ROP rats, which also had significantly shorter axial lengths. These data reveal the ROP rat to be a novel and potentially informative approach to investigating physiological mechanisms in myopia in general and the myopia peculiar to ROP in particular.

## 1. Introduction

Retinopathy of prematurity (ROP) presents as abnormal retinal blood vessels in an ophthalmoscopic exam of the premature infant. Evidence indicates that the appearance of the abnormal retinal blood vessels in ROP is instigated by changes in the neural retina [1, 2]. In addition, infants born prematurely are at increased risk for developing a range of structural ophthalmic sequelae including impaired ocular growth and increased incidence and magnitude of refractive error, particularly myopia [3, 4]. Myopia is a mismatch between the light-focusing power of the anterior segment and the axial length of the eye in which the visual image comes to a focus in front of the retina. Myopia is therefore typically associated with longer-than-average eyes [5]. Paradoxically, in ROP myopia the eye is usually small [3, 4, 6–14]. These common, clinically important ROP outcomes—vascular, neurologic, and structural—are likely interrelated.

Visual impairment, with a basis in the neural retina, is commonly found in subjects with a history of ROP, even when the vasculopathy was mild [15–20]. Specifically, psychophysical dark-adapted and increment threshold functions obtained in ROP subjects show higher *eigengrau* (optic nerve signaling in darkness [21]) values [22, 23], likely a consequence of disorganized [24] or fewer photoreceptors [25]; subtle differences in the vascular supply may also be at play [25]. Notably, the psychophysical changes are most marked in ROP subjects with high myopia while such abnormalities are *not* found in similarly myopic control subjects [26]. Electroretinographic (ERG) studies of retinal function also reveal deficits that are significantly associated with early myopia [27]. Defects in “ON signals” are associated with anomalous eye growth [28] and are abnormal in the ERGs of eyes with a history of ROP [26, 29]. For instance, there is evidence of a depressed postreceptor ON signal in the multifocal ERG (mfERG) responses of myopic children with a history of mild ROP that is not found in myopes with no ROP [29]. Taken together, the psychophysical and ERG data from ROP and control subjects with and without myopia imply that deficits in retinal function in ROP are not explained by myopia alone [27]. Despite refractive errors being collectively the most common sequela of ROP and therefore of high clinical importance, the mechanisms underlying the altered eye growth remain poorly understood. No doubt this is partly for lack of a relevant animal model.

The retina controls eye growth and refractive development [30, 31]. Evidence from simian eyes [32, 33] strongly indicates that it is the peripheral retina, in particular, which is most important to these processes (although the evidence in humans is weaker [34]). Notably, the peripheral retina is avascular in ROP. The avascular peripheral retina must have altered function, and thus it should not be surprising that the vasculopathy which clinically characterizes ROP is also strongly associated with altered eye growth and ametropia [8–11, 35, 36].

Rat pups exposed to a clinically relevant [37] alternation of relatively high and low oxygen during the first weeks after birth develop a retinopathy that models human ROP [37, 38]. This oxygen-induced retinopathy (OIR) represents a convenient *in vivo* model in which to study ROP that has been widely adopted, the so-called “ROP rat.” The ROP rat's vascular abnormalities include an avascular peripheral retina and neovascularization [39–41], as in human ROP [42]. Also as in human ROP, retinal function is persistently abnormal [43–52]. Ocular structures have been studied in normal rats but have received only limited attention in ROP rats. Whether or not the ROP rat mimics the ametropias common to human ROP remains to be determined, although, in a histological study of young ROP rats with active disease, the OIR eye was found to be smaller with a relatively shorter anterior segment than the room-air-reared (RAR) rat's eye [53], as is again the case in human ROP [3, 4].

Manganese-enhanced magnetic resonance imaging (MRI) of the retina in RAR rats finds that it thins following a posterior-to-periphery gradient; in contrast, ROP rats' retinae are more uniform in thickness [47, 52]. As the eye grows, the peripheral retina, posterior to the iris and anterior to the equator (where the ocular muscles attach), may “stretch” to pave a larger area. A failure of the eye to grow in this fashion would be reflected in the more uniform retinal thickness of the ROP rat. Calcium channel activity in the postreceptor retina is supernormal during active disease in ROP rats and decreases as the vasculature matures [47, 52]. Likewise, relative to posterior retina (and to any region of normal retina), oxygen tension is lower in the avascular periphery of OIR eyes [54]. Changes in autoregulation of retinal oxygenation persist long after active disease has resolved [55]. That the retina and its circulation are rendered persistently dysfunctional in ROP rats might limit its ability to mediate emmetropization by regulating the growth of the sclera.

To be both small and myopic, the anterior segment of an eye must be of substantially higher-than-normal dioptric power. The hyaloidal vasculature that supplies the developing lens is present in the prematurely born infant [56, 57] and persists, much engorged, in ROP [57, 58]. MRI reveals that the same is true in the ROP rat [59]. Furthermore, in the RAR rat, the regression of the hyaloid is well coordinated with the development of other ocular structures, such as the vitreous chamber and crystalline lens; in the ROP rat, growth of ocular structures and hyaloidal regression proceed in a less-coordinated fashion [59]. Prolonged hyperemia of the anterior segment might lead to changes in the shape and thickness of the lens and cornea that, combined with a shorter anterior segment length, would lead to increased refractive power. Thus, it is plausible that the ROP rat models the peculiar ametropia common to ROP: small-eyed myopia.

In summary, the refractive state of the eye depends upon the refractive indices and curvatures of its various media and their spatial relationships to each other and the retina, and there is plentiful reason to suspect that the development of the optic media is altered in ROP. Assessing the refractive state of small eyes using traditional approaches such as retinoscopy is notoriously difficult. Further, the so-called “small-eye artifact” is well documented [60] but remains problematic and poorly specified [61]. Some modern approaches, like wavefront sensing and automated photorefracting [28, 62], are less variable but are not immune to the artifactual distortion of small-eye refractions. Furthermore, those methods do not provide details about the relative contributions of the cornea and lens or their relative positions. Advanced imaging techniques like MRI permit inspection of these surfaces noninvasively and, importantly, in their intact state (something that cannot be achieved *ex vivo*) and do so *simultaneously*. They are also theoretically immune to artifacts of eye size wherein the retinal origin of reflections is problematic [63]. Other approaches, such as high-frequency ultrasound, optical coherence tomography, and multiple-wavelength interferometry may (especially in the future) be able to produce biometry of resolution comparable to today's MRI. A high-quality schematic eye for the adult rat is available [64] that provides the refractive indices of the cornea and lens. Allowing for a number of assumptions (detailed below), the schematic eye provides a framework from which the refractive status of *any *rat eye can be estimated from an MRI of the globe. In this study, structural measurements were obtained from MRIs of immature and adult ROP and RAR rat eyes and referenced to the previously published schematic eye to calculate refractive state (*℞*). The calculations suggest that the adult ROP rat is, indeed, characterized by small-eyed myopia. A comparison between albino (Sprague-Dawley) and pigmented (Long-Evans) strains is also included.

The ROP rat can provide insights into refractive development that cannot be observed from traditional myopia models (e.g., form deprivation [5, 65]) wherein the eye is large and can provide a basis for biochemical (genetic, protein, etc.) investigations into the most common and least studied clinical sequela of ROP: refractive error.

## 2. Materials and Methods

### 2.1. Subjects

Sprague-Dawley albino and Long-Evans hooded rats were studied. As described elsewhere in detail [46], OIR was induced in ROP rats by placing pups and dams in an OxyCycler (Biospherix Ltd., Lacona, NY, USA) and exposing them to alternating 24-hour periods of 50% and 10% oxygen from postnatal day (P) 0, the day of birth, to P14 [39]. This “50/10 model” reliably produces peripheral neovascularization and increased tortuosity of the posterior retinal arterioles [39, 44, 46, 47, 66]. RAR rats served as controls. The Sprague-Dawley 50/10 model rat is considered the canonical 50/10 model [41].

### 2.2. Magnetic Resonance Imaging

The present images were previously collected and analyzed as part of our ongoing MRI studies of the neural retina. Most of these images were used to generate previous reports (summarized in Berkowitz and Roberts, 2010, [51] and Berkowitz et al., 2011 [52]). The imaging methods, briefly described here, are detailed therein and elsewhere: after rats were anesthetized with freshly prepared 36% urethane IP (~0.083 mL/20 g; Sigma-Aldrich, Milwaukee, WI, USA), T_1_-weighted spin-echo images were obtained on either a 4.7 T Bruker Avance system (repetition time, TR = 350 s; echo time, TE = 16.7 ms; Sprague-Dawley images) or a 7 T Bruker ClinScan system (TR = 1 s; TE = 13 ms; Long-Evans images) using a 1 cm diameter surface coil placed around the left eye. A cross-sectional image of the left eye was collected as a single, 600 *μ*m thick slice passing through the optic nerve head and pupil center. To be deemed suitable for analysis, both the pupil and optic nerve needed to be clearly visible in all images, indicating negligible deviation from the central axis of the eye. In-plane axial spatial resolution (i.e., along a line passing though the cornea, pupil, lens, and central retina) was always ≤25 *μ*m/pixel width.

### 2.3. Image Analyses

All images were analyzed using a custom-developed MATLAB (The Mathworks, Inc., Natick, MA, USA) program. First, each MR image was rotated so that the plane of the *ora serrata* was parallel with the horizontal axis. Two intensity plots were then obtained along the lines passing (a) through the pupil center and the optic nerve head (ONH) and (b) through the plane of the *ora serrata*. Then, each image was thresholded into a binary (black and white) image for segmentation of the ocular structures from the fluid bodies (e.g., air and aqueous and vitreous humors). From the intensity plots and segmented images, the positions of the ocular media and their curvatures were, respectively, determined.

#### 2.3.1. Positions of the Ocular Media

**Figure 1:**
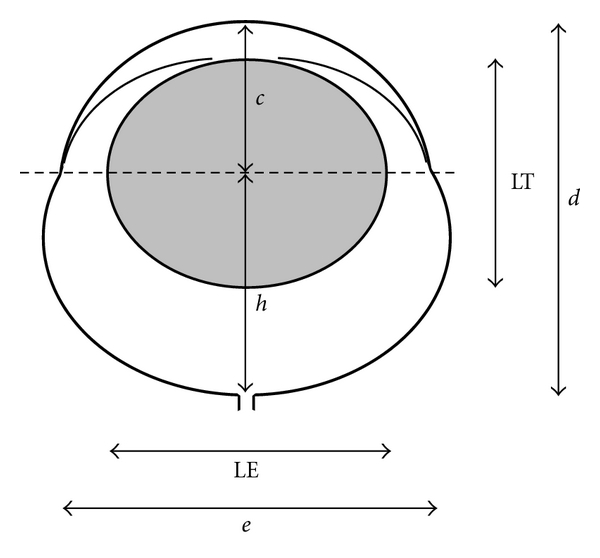
Schematic diagram showing the ocular dimensions obtained from the MRI. *d:* axial length; *c:* anterior segment length; *h:* posterior segment length; *e:* equatorial diameter at the plane of the *ora serrata *(*dashed line*); LT: lens axial thickness; LE: lens equatorial diameter.

Measures of ocular dimensions were determined from the peaks and troughs on the first derivative of the intensity plots assuming that the edges of the ocular surfaces corresponded to the most rapid changes in intensity. The following biometric parameters, based upon Robb's [67], were thus obtained (Figure 1): (1) the “diameter” between the apex of the cornea and the posterior pole of the retina (axial length, *d*), (2) the portion of the axial length from the apex of the cornea to the plane of the *ora serrata* (anterior segment length, *c*), (3) the remaining distance from the *ora serrata* to the posterior pole (posterior segment length, *h*), (4) the diameter of the eye along the plane of the *ora serrata* (equatorial diameter, *e*), (5) lens thickness (LT), and (6) equatorial diameter (LE).

#### 2.3.2. Measurements of Curvature

**Figure 2:**
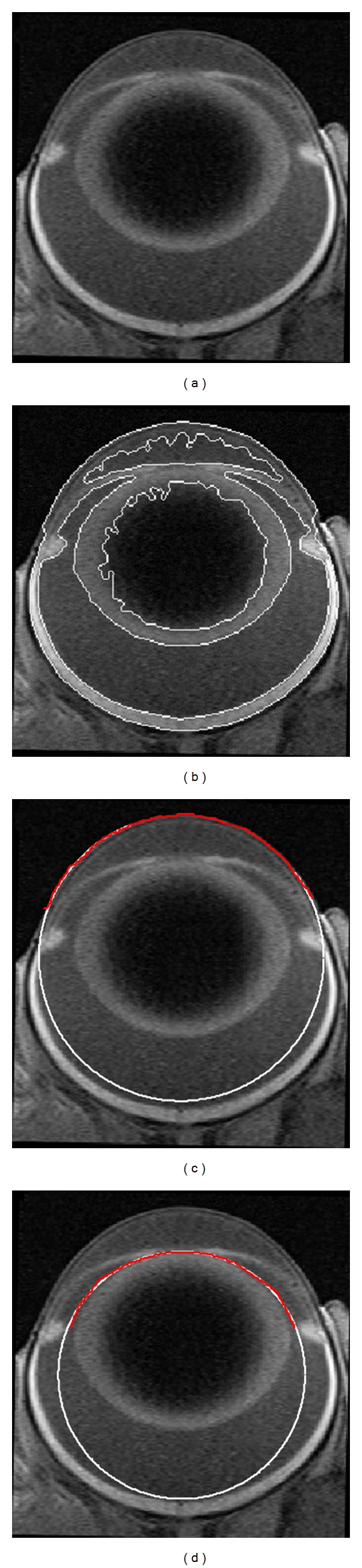
(a) MR image of a Long-Evans control rat P50. (b) The same image with the detected edges. (c) Circle (*white*) fit to the edge data of the anterior cornea surface (*red*). (d) Circle (*white*) fit to the edge data of the anterior lens surface (*red*).

Edge detection on the binary image was performed using the Canny method available in MATLAB. The subset of edge data from the relevant ocular surfaces (cornea, lens, and retina) was selected by the operator (TYPC) on the MR image from the superset of detected edges (Figure 2(b)). Occasionally, edge detection on the full anterior lens surface was hindered by the iris; in these cases the operator added edge data by tracing the obscured region of the surface manually and removed the iridic edge from further analysis. Following segmentation, the anterior cornea surface, anterior lens surface, and posterior lens surface were identified by the operator and circles were fitted through the respective edge data using a least-squares approach (Figures 2(c) and 2(d)), providing radius of curvature parameters for the optical media. These measurements were validated by a second reviewer (DB) for a large subset of images using a less-automated approach, developed in R [68], which yielded nearly identical results (not shown).

### 2.4. Calculation of Refractive State

**Figure 3:**
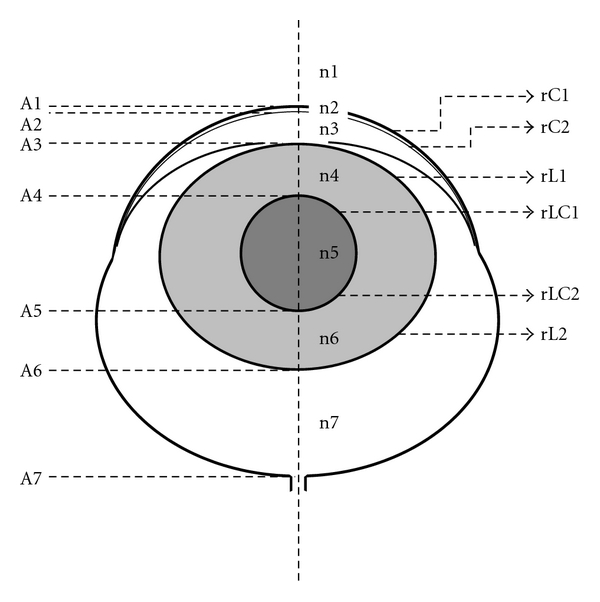
Schematic rat eye model with main parameters needed for refractive state estimation (see Table 1). A1 (corneal surface) through A7 (vitreoretinal border) are distances along the horizontal dashed line; 130 *μ*m was added to A7 to approximately account for the postreceptor retina contribution. If A1 is set to 0 mm from corneal surface and numbers increase from top to bottom, then rC1, rC2, rL1, and rLC1 are positive, while rLC2 and rL2 are negative.

The refractive state of the rat eye was computed based on the ocular dimensions and the curvatures of its various refractive surfaces measured from the MR images using either the core lens model (Figure 3) of Hughes (1979) [64] or assuming a homogeneous lens. Refractive indices of all ocular media were adopted from Hughes as constants [64]. The parameters used and the values employed, or that they were directly measured or derived, are given in Table 1 (parameters not used in the homogeneous lens model are marked as “not applicable” by “NA”).

The curvature of the anterior corneal surface (rC1) was measured as described above. However, edge detection of the posterior corneal surface proved unsatisfactory. Thus, in the final analysis, measurements of the corneal thickness (A2 − A1) and the curvature of the posterior corneal surface (rC2) were not made but were instead derived for each MR image based on the ratio of (A2 − A1)/(A7 − A1) and rC1/rC2 obtained from Hughes' [64] study, respectively. The whole lens thickness (LT = A6 − A3) and curvatures of the anterior and posterior lens surfaces (rL1, rL2) were measured directly in the study. However, for the core lens model, the core lens thickness (A5 − A4) was scaled linearly for each MR image based on the measured LT, and the ratio of core thickness to lens thickness, (A5 − A4)/LT, described by Hughes. Since the lens core of this model is spherical [64], the radii of curvature for the anterior and posterior core lens surfaces (rLC1, rLC2) were, respectively, computed as plus and minus half of the derived core lens thickness.

The refractive state of the eye was derived by calculating and combining the dioptric powers and the principal points of the cornea and lens components following the method of Southall [69] and the notation in Hughes [64]. The complete formulae needed to satisfy (1) through (4), below, are given in the Appendix.

The power of the cornea (FC) was calculated as

(1) FC=F1+F2−c1·F1·F2,

where F1 and F2 are the respective powers (D) of the anterior and posterior surface of the cornea, and c1 is the reduced interval (m) between them.

The power of the lens was calculated in two ways, assuming either a homogenous lens (FL_hmgns_) or using the core lens model (FL_core_) of Hughes [64]. For the core lens model,

(2) FL=F(3,4)+F(5,6)−s·F(3,4)·F(5,6),

where F(3,4) and F(5,6) are the respective powers of the anterior and posterior lens system, including half of the core in each, and s is the reduced interval between the anterior and posterior lens system. When the homogenous lens was assumed, the terms relating to the core lens (F4 and F5) were omitted and the equation for FL_hmgns_ adjusted accordingly (including changing the reduced interval to be that between the anterior and posterior lens surface; see the appendices).

Hughes' [64] formula was used in the final determination of *℞*. First, the refracting power of the whole eye was derived, for both the homogeneous (FE_hmgns_) and core lens (FE_core_) models, as

(3) FE=FC+FL−cE·FC·FL,

where cE is the reduced interval between the cornea and lens components (FC, FL). Second, *℞* was derived (for both lens models) by

(4) $$℞=\frac{\text{n}7}{\left(\text{A}7-\text{A}1\right)-\text{A}1\text{H'}}-\text{FE}.$$

In (4), A1H′ is the distance (m) between the anterior cornea surface (A1) and the second principal point of the eye (H′).

### 2.5. Data Analyses

*℞* was evaluated by analysis of variance (ANOVA). Because significant changes in *℞* were detected between levels of factors, the sources of the changes were explored by evaluating the dioptric powers of the cornea (FC_core_) and lens (FL_core_) in a second ANOVA and the ratio of anterior to posterior depth (*c*/*h*) in a third ANOVA. To determine if axial length was affected by ROP, *d *was evaluated in a fourth ANOVA. To detect changes in the gross shape of the eye and lens, the ratio of axial length to equatorial diameter (*d*/*e*) and lens thickness and diameter (LT/LE) were also, respectively, evaluated in a fifth and sixth ANOVA. *Post hoc* testing was performed using *t*-tests corrected by the Bonferroni method. The acceptable type-1 error rate (*α*) for all tests was 5%, but because the parameters of the multiple analyses were not likely independent, significance for each ANOVA was set to a more conservative *P* ≤ 0.01.

## 3. Results and Discussion

**Figure 4:**
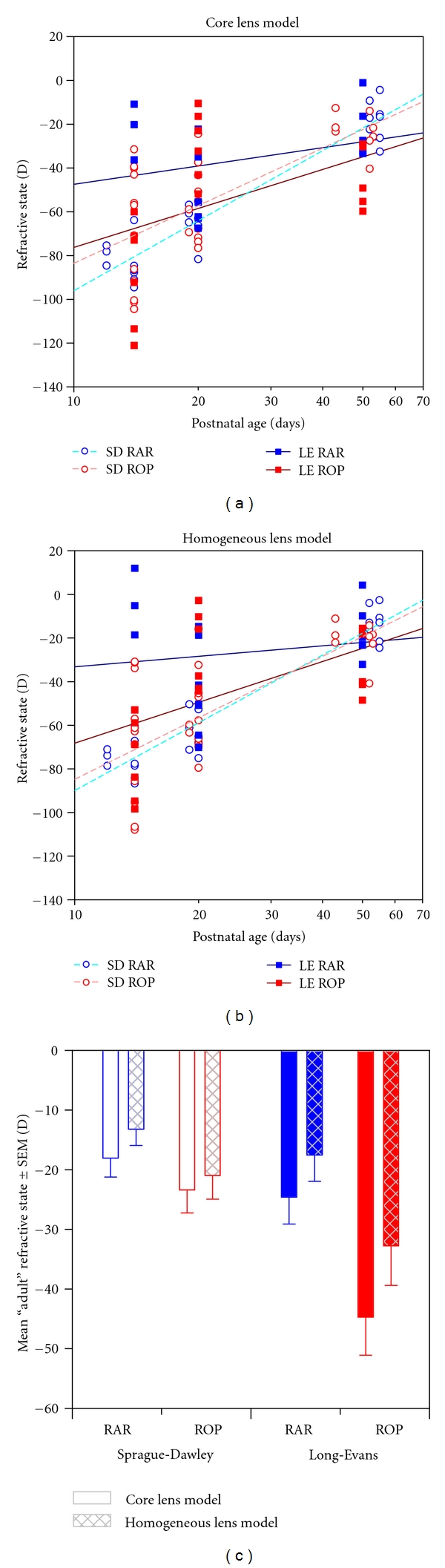
Measurements of refractive state, *℞*. (a) *℞* measured using the core lens model (*℞*_core_). Lines are log-linear regressions through Sprague-Dawley (*light dashed*) and Long-Evans (*dark solid*) ROP (*red*) and RAR (*blue*) rats' data. (b) *℞* measured using the homogeneous lens model (*℞*_hmgns_). Lines as in (a). (c) *℞* at ~P50, an “adult” age.

Ninety images were suitable for analysis, 56 from Sprague-Dawley rats (24 RAR, 32 ROP) and 34 from Long-Evans rats (17 RAR, 17 ROP). *℞* was estimated for each animal, and the results are plotted in Figures 4(a) and 4(b). As shown therein, data were collected at approximately postnatal day (P) 14 (at the end of the induction of retinopathy [39]), at ~P20 (when the disease is active and neovascularization is present [44, 46, 51, 54, 55]), and at ~P50 (an “adult” eye [70–73] with “normal” vasculature [53]). Analysis of these *℞* data by lens model × age × group × strain repeated measures (lens model) ANOVA revealed significant main effects for all factors and several significant interaction effects, as discussed below.

### 3.1. Core versus Homogenous Lens Model

The *℞* results obtained using the core (Figure 4(a)) and homogenous (Figure 4(b)) lens models were highly correlated (*r* = 0.94). As shown in Figure 4(c), which plots respective *℞* means (±SEM) for ROP and RAR Sprague-Dawley and Long-Evans rats from the ~P50 data, the homogenous model tended to yield relatively less myopic “refractions” (*F*(model) = 59.8, *df *= 1,78, *P* < 10^−10^): in every case the homogenous lens model predicted less average myopia (although not always so in individual rats). There was not, however, a significant model × group interaction (*F*(model × group) = 2.55, *df *= 1,78, *P* = 0.114), so that interpretation of the ROP *versus* RAR data does not depend significantly upon the lens model selected. Hughes preferred the core lens model since it produced refractive estimates closer to his (roughly emmetropic) assessments of the refractive state of the adult rat eye [74]; in the present study, however, it is the homogenous lens model that produced *℞* estimates closest to emmetropia. Careful reevaluation of Hughes' values (his Table  2 [64]) using his core lens model yields slightly hyperopic refractive estimates for his rats. Therefore, the discrepancy between Hughes' data and the data in the present study may be due to the age of the rats, which were 115 to 130 days old in that study. Furthermore, advances in noninvasive imaging techniques may soon permit analysis of the lens gradient in the rat *in vivo* [75], improving estimates of refractive state. In any event, it is likely that the normal rat is approximately emmetropic throughout its adult life [61, 76].

### 3.2. Emmetropization

There was strong evidence of emmetropization (Figures 4(a) and 4(b)) in RAR and ROP Sprague-Dawley rats (*dashed light blue and light red lines*) as well as ROP Long-Evans rats (*dark red lines*) resulting in a highly significant effect of age (*F*(age) = 32.6, *df *= 2,78, *P* < 10^−10^). On the other hand, the change in *℞* in normal Long-Evans rats (*dark blue lines*), who were relatively emmetropic at ~P14 and ~P20, and remained similarly myopic ~P50, was significantly less (*F*(age × group) = 12.2, *df *= 2,78, *P* < 10^−4^). Two interesting elements of the emmetropization process revealed in these data are discussed below.

First, in the *℞* data of normal, RAR rats, the young Sprague-Dawley rats appeared highly myopic and became relatively less so over time. In this respect, the Sprague-Dawley rats differed significantly from the Long-Evans rats (*F*(strain) = 12.0, *df *= 1,78, *P* = 0.001). Retinoscopic measurements of refractive development in the pigmented (Brown Norway) rat have not shown systematic changes with age [77], consistent with the data from the pigmented rats herein, but no attempt has (to the authors' knowledge) been made to monitor the refractive development in the albino rats' eye. Nevertheless, the standard process of emmetropization—progression from hyperopia to emmetropia [78]—is the obverse of the progression found herein in the RAR Sprague-Dawley rats.

Second, *post hoc* testing revealed that in the adult animals (~P50), ROP rats were significantly more myopic than RAR controls (*P* = 0.007). This was despite the fact that the Sprague-Dawley ROP rats were *less *myopic at ~P14 than the RAR rats of the same strain. That is, regardless the amount of ametropia in each group at ~P14, emmetropization left both the Sprague-Dawley and Long-Evans ROP rats myopic at ~P50, relative to strain-matched RAR controls (*F*(age × strain × group) = 11.7, *df *= 2,78, *P* < 10^−4^).

### 3.3. Severity of Ametropia and Strain

By retinoscopy, many (if not all) strains of rats appear hyperopic [77, 79]. Hooded-rats, like the Long-Evans, appear approximately 5–15 D more hyperopic than Sprague-Dawley rats. Estimates of refractive state by visually evoked potential (VEP) found rats are, in fact, more emmetropic than retinoscopy indicates [61]. Nevertheless, in the data from the adult rats in the present study, *℞* was correspondingly more relatively myopic in the Sprague-Dawley than Long-Evans rats (Figure 4(c)). Indeed, the magnitude of ametropia in RAR Long-Evans rats was similar to that in the ROP Sprague-Dawley animals (i.e., the second and third sets of columns in Figure 4(c) appear comparable).

Thus, by retinoscopy and now by MRI “refractions,” the albino strain appears more emmetropic than the pigmented one. However, caution in interpretation of this finding is urged because the *direction* of the pigmented rats' measured ametropia is opposite using each approach: more hyperopic by retinoscopy and more myopic herein. Since the rat eye is so powerful, tiny errors, such as those from rounding, in the refractive indices used in the calculations of *℞* would result in large changes in the estimate of the refractive power of the eye. Indeed, it is quite plausible that pink- and black-eyed animals' optical media would refract light somewhat differently [80]. For these reasons, comparisons of strain-matched experimental groups may prove most reliable in future studies. Nevertheless, in the present dataset, OIR caused a larger shift toward myopia—across every age—in Long-Evans than Sprague-Dawey rats (*F*(group × strain) = 11.1, *df *= 1,78, *P* = 0.001).

### 3.4. Biometric Bases of ROP Myopia

**Figure 5:**
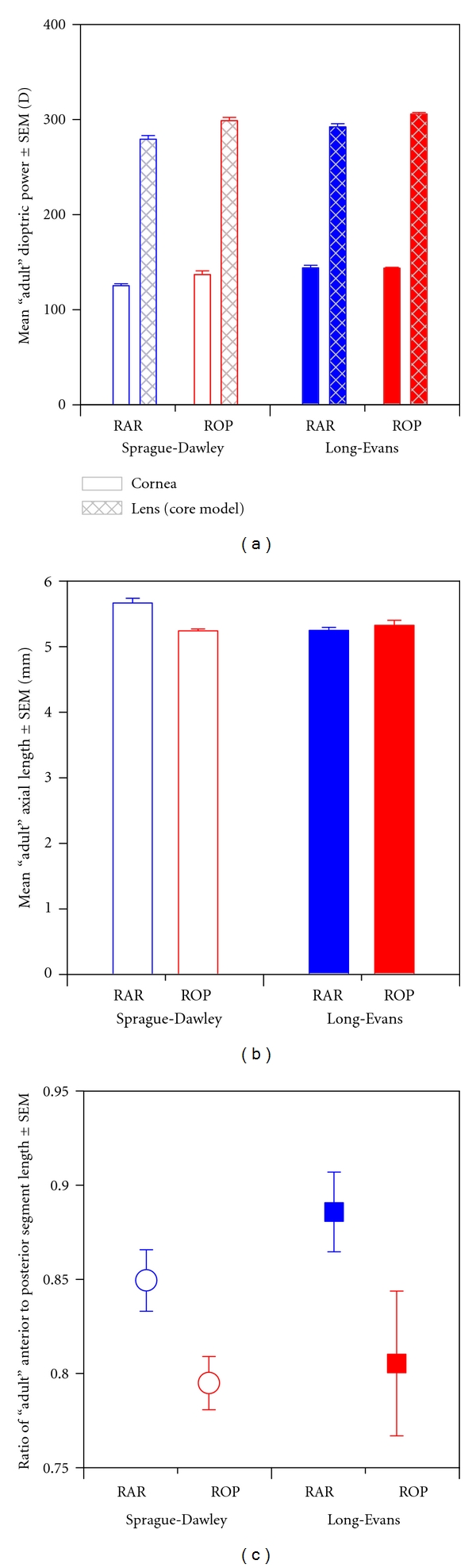
Biometric bases of *℞* at ~P50. (a) Dioptric power of the cornea and lens. (b) Axial length (*d*). (c) Quotient of anterior segment length (*c*) divided by posterior segment length (*h*).

Human myopia of prematurity, exacerbated by ROP, persists into adulthood. On average, relative to myopic adults born full term, adults with the same degree of myopia and a history of ROP have eyes with shorter axial length, increased corneal curvature, increased lens thickness, and shallow anterior chamber depth. Amongst these features, the increased corneal curvature is most responsible for the myopia [13]. In the human eye, despite the fact that the lens is a more powerful convergent surface than the cornea, the cornea contributes approximately two-thirds of the total refracting power to the eye (~43 D) because the gelatinous aqueous humor provides a weaker index of refraction than air [81]. As shown in Figure 5(a), which plots the contributions of the cornea (FC) and lens (FL_core_) in the ~P50 ROP and RAR Sprague-Dawley and Long-Evans rats, the proportion is reversed: the rat cornea contributes only about a third of the total refracting power to the eye. Thus, changes to the lens might be very important in ROP rat myopia.

Indeed, the results of media × group × strain repeated measures ANOVA (media: cornea *versus* lens) in ~P50 rats revealed that the cornea contributed significantly less power to the eye than the lens (*F*(media) = 6,930, *df *= 1,24, *P* < 10^−31^). In ROP rats, the total dioptric power of the cornea (FC) and lens (FL_core_) was higher than that in RAR controls (*F*(group) = 22.1, *df *= 1,24, *P* < 10^−5^). The Sprague-Dawley rats had less powerful media than the Long-Evans rats (*F*(strain) = 23.1, *df *= 1,24, *P* < 10^−5^). And indeed, the increase in the power of the lens in ROP was greater than that in the cornea (*F*(media × group) = 8.19, *df *= 1,24, *P* = 0.009); in the Long-Evans rats, in fact, corneal power did not change at all in ROP (*P* = 0.91).

### 3.5. Paradoxical Myopia?

As stated earlier, the myopia characteristic of prematurity and ROP is a peculiar one in that a history of ROP is also associated with short axial length. The axial length data (*d*) were analyzed at ~P50 by group × strain ANOVA. As shown in Figure 5(b), short axial length is also a feature of the ROP rat (*F*(group) = 9.34, *df *= 1,24, *P* = 0.005) but only the albino (*F*(group × strain) = 20.2, *df *= 1,24, *P* < 10^−4^) which normally had a larger eye (*F*(strain) = 8.31, *df *= 1,24, *P* = 0.008).

Furthermore, as indicated in Figure 5(c) and confirmed by group × strain ANOVA, the ratio of anterior-to-posterior segment depth (*c*/*h*) was significantly reduced at ~P50 in both the Spague-Dawley and Long-Evans ROP rats (*F*(group) = 10.3, *df *= 1,24, *P* = 0.004), as it is in human ROP myopia [3, 4].

### 3.6. Changes in Eye Shape

**Figure 6:**
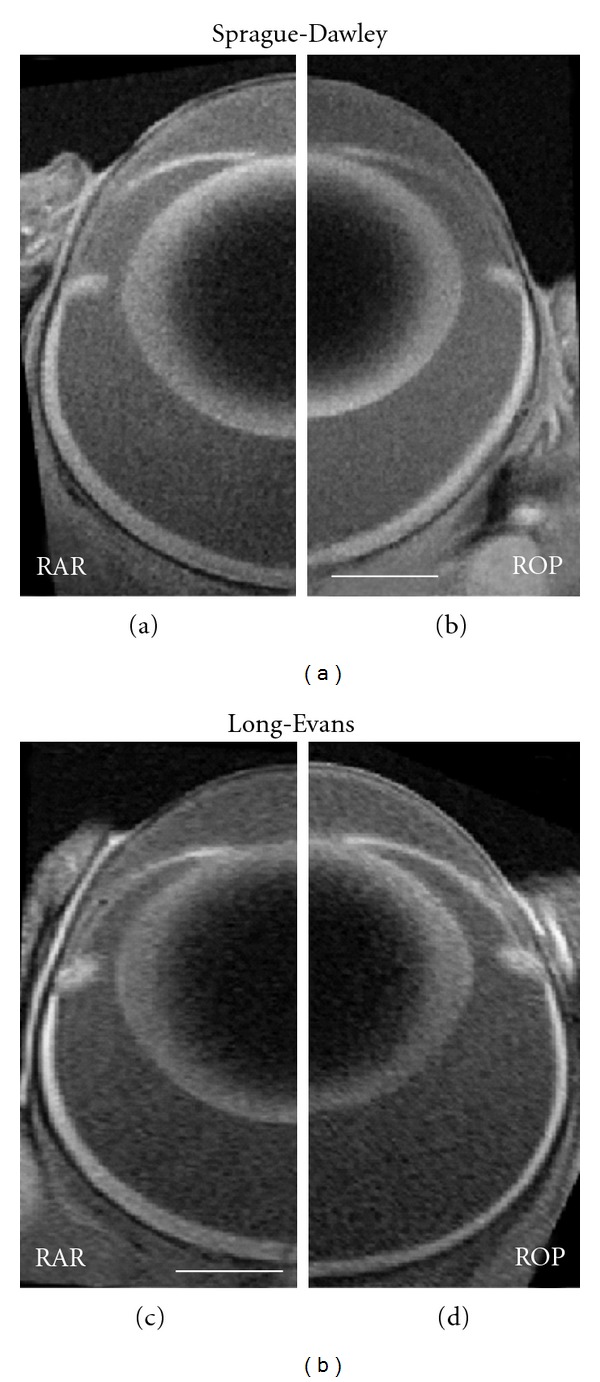
MR images obtained in ~P50 Sprague-Dawley RAR (a) and ROP (b) rats and Long-Evans RAR (c) and ROP (d) rats. All images are displayed at the same magnification (scale bars are 1 mm). The ROP rat images (b, d) are mirrored to eliminate nasal-temporal asymmetries, if any. Note that the ROP rat eyes are characterized by steeper corneae and relatively reduced anterior segment depths. Note also that the Sprague-Dawley ROP rat has a short axial length.

The subjective appearance of the ROP eyes was occasionally heteroclitic beyond just the noted changes to the refractive surfaces of the eye and their spatial interrelations (Figure 6). This might be the case if the ROP eyes' failure to elongate along the visual axis was not matched by an equivalent failure to expand along the perpendicular axis, thus creating a “fatter” eye. To test for changes in the proportions of the eye, the ratio of axial length over equatorial diameter at the plane of the *ora serrata* (*d*/*e*) at ~P50 was evaluated in a group × strain ANOVA. No significant effect of group was found. In addition, test of the ratio of lens thickness over lens equatorial diameter (LT/LE) likewise detected no significant effect of OIR.

### 3.7. Methodological Limitations

The absolute refractive measurements based on MRI appear reasonable (*e.g.*, in adults) but are, of course, limited by the modeling assumptions. That is, an MRI “refraction” of *plano ℞* does not necessarily indicate a truly emmetropic eye. That said, comparison of refractive state estimates by retinoscopy and VEP [61] indicate that it is the outer-middle retina that accounts for the retinoscopy reflex and not the inner limiting membrane (ILM) as has often been suggested [60]. The measurement of retinal position (A7; Table 1) in the present study was at the vitreoretinal boundary; therefore, 130 *μ*m was added to A7 to make these MRI “refractions” more comparable to those obtained by retinoscopy. To estimate *℞* ILM from these data, it is therefore necessary to add ~10 D (i.e., *less* myopia) to the results shown in Figure 4. As earlier discussed, the normal rat is probably close to emmetropic [76] (although slightly hyperopic [61] and slightly myopic [82] measures have both been reported for the murine eye) in adulthood. Thus, this roughly 10 D correction *may* better align estimates of refractive state obtained *via *this MRI procedure with those obtained by other techniques.

Note that neither errors in the refractive indices used herein nor the particular selection of retinal position in the calculation of *℞* should impact much the *relative* relationships between the refractive states derived for the rats in this study: the calculations in all animals would be similarly impacted by such systematic errors. Future comparisons between this method and other techniques (retinoscopy, photorefraction, wavefront sensing, electrophysiology, etc.) may reveal what the necessary correction is (if any) to reach agreement between sundry techniques.

### 3.8. Relationship to Human ROP

The induction of experimental ROP lasts through the first 14 days of the rat's life, and a 50-day-old rat may be roughly equated to an adolescent or young-adult human. The equivalent disturbance in the human would last at most a couple of months. Thus, the time to recover from the original, oxygen-induced insult to the eye would be only about two-thirds or three-quarters of the lifetime (to date) for the rats at ~P50, but ~99% of the lifetime for the equivalent young-adult human, perhaps an important difference. However, to achieve parity in this respect, the rat would need to be tested more than 1,000 days after the induction of retinopathy, a span longer than the typical life of a lab rat. Evaluation of refractive state in rats older than P50 might nevertheless provide valuable information. That said, to date the ROP rat has been mostly considered a model of retinal neovascularization. At least over the timeframe included in this study, the ROP rat seems also to be a novel model of myopia.

A further difficulty is that the OIR consistently models a moderate ROP, neither particularly severe (retinal detachments are not noted in the literature on this ROP rat model, although they are in others [83, 84]) nor particularly mild (marked NV occurs in 100% of animals). As detailed in the Introduction section, in human ROP the severity of the vasculopathy is related to the severity of ametropia but leads to greater incidences of both myopia *and* hyperopia, with myopia predominating. The range of disease severity in human eyes is much broader than in the model and, in the most severe cases, is generally treated using laser ablative therapy. The consequences of treatment on the present outcomes in the ROP rat were not investigated. Nevertheless, two multicenter trials for the treatment of severe ROP, CRYO-ROP and ETROP, concluded that ROP treatment does not itself influence refractive outcomes [85, 86]. That said, in addition to ROP severity, birth weight and degree of prematurity may be additional, independent risk factors for myopia [35, 36], neither of which are factors accounted for in the rat model. Slow postnatal weight gain, which is increasingly recognized as an important prognostic of ROP severity in both human ROP [87, 88] and rodent OIR models [89, 90], *was* controlled for in large part by supplementing litter sizes to 12–15 pups; these “expanded litters” (typical litter size is 10–12) increase competition for milk supply [91] and express more severe retinopathy [89]. Note that even after matching litter size, ROP rats weigh approximately half as much as age-matched RAR rats at the conclusion of the oxygen exposure regimen, a gap they reduce but never close.

### 3.9. Final Thoughts

The ROP rat models well the myopia peculiar to premature birth and which is exacerbated by ROP: short axial length, increased corneal power and lens power, and proportionally shallow anterior segment. The albino Sprague-Dawley strain, in particular, appears to model *all *of these characteristics, while the hooded Long-Evans strain suffers from a more exaggerated ametropia but no change in axial length. Several developmental features of the ROP rat's eye may underpin these phenomena: First, the decreased axial length may be a consequence of an (especially) dysfunctional peripheral retina consequent to prolonged hypoxic ischemia from a failure of normal peripheral vascularization. Second, the much increased lens power may be consequent to prolonged hyperemia of the anterior segment mediated by a persistent, engorged, and unregulated hyaloid. Third, the normally exquisite mediation of emmetropization may be lacking due to retinal dysfunction as well as a poorly regulated ionic retinal *milieu* [92], an imbalance [51] of which perhaps travels the uvea or vitreous from the retina to anterior segment [93]. Fourth, alterations in retinal, vitreal, or uveal levels of other paracrine signaling molecules, such as dopamine or nitric oxide, are also plausible [5, 94–96]. Further experiments are needed to ascertain if these and other factors are indeed at play in this sight-threatening condition.

Regardless of the underlying mechanisms, the short-eyed myopia found in the present study is distinct from other myopia models in that it accurately models the clinical myopia of prematurity. Study of the ROP rat may therefore provide insights into ocular development difficult or impossible to obtain using traditional models such as the chick or monkey with occluded vision. Furthermore, the correlation between optical and neurovascular abnormalities implies that treatments that result in less severe myopia will also be beneficial to the underlying retinal pathology. The method described in this paper, specifically the use of the noninvasive MRI, makes for ready translation from animal models to human patients.

## Figures and Tables

**Table 1:** Parameters for refractive state estimation in rat eye.

Category	Parameters	Symbol	Homogeneous lens model	Core lens model
Refractive indices*	Air	n1	1.000	1.000
	Cornea	n2	1.380	1.380
	Aqueous and vitreous humors	n3, n7	1.337	1.337
	Lens cortex	n4, n6	NA	1.390
	Lens core	n5	1.683	1.500

Axial positions (m)	Anterior cornea surface	A1	Measured from MRI	Measured from MRI
	Posterior cornea surface	A2	Scaled^†^	Scaled^†^
	Anterior lens surface	A3	Measured from MRI	Measured from MRI
	Anterior core lens surface	A4	NA	Scaled^†^
	Posterior core lens surface	A5	NA	Scaled^†^
	Posterior lens surface	A6	Measured from MRI	Measured from MRI
	Retina	A7	Measured from MRI + 130 *μ*m	Measured from MRI + 130 *μ*m

Radii of curvature (m)	Anterior cornea surface	rC1	Measured from MRI	Measured from MRI
	Posterior cornea surface	rC2	Scaled^†^	Scaled^†^
	Anterior lens surface	rL1	Measured from MRI	Measured from MRI
	Anterior core lens surface	rLC1	NA	(A5 − A4)/2
	Posterior core lens surface	rLC2	NA	(A5 − A4)/2
	Posterior lens surface	rL2	Measured from MRI	Measured from MRI

Dioptric powers (D)	Cornea	FC	Equation (1)	Equation (1)
	Lens	FL	Reduced equation (2) (FL_hmgns_)	Equation (2) (FL_core_)
	Whole eye	FE	Equation (3) (FE_hmgns_)	Equation (3) (FE_core_)
	Refractive state	*℞*	Equation (4) (*℞*_hmgns_)	Equation (4) (*℞*_core_)

*Refractive indices are adopted from Hughes (1979).

^†^Parameters were obtained by scaling linearly to the values obtained from Hughes' study.

## Appendices

The following formulae provide for all calculations needed to satisfy (1)–(4) in the text, also reprinted below. They are derived from Southall [69], wherein fuller explanations can be found. Parameter values were either taken from Hughes [64] or were measured in the MR images, as indicated in Table 1.

### A. The Cornea

Refracting power of the anterior cornea surface

(A.1) $$\text{F}1=\frac{\text{n}2-\text{n}1}{\text{rC}1}.$$

Refracting power of the posterior cornea surface

(A.2) $$\text{F}2=\frac{\text{n}3-\text{n}2}{\text{rC}2}.$$

Reduced interval between the two surfaces

(A.3) $$\text{c}1=\frac{\text{A}2-\text{A}1}{\text{n}2}.$$

Refracting power of the cornea system (FC) is as shown in (1).

### B. The Lens

(B.1) The Anterior Lens System Refracting power of the anterior lens surface

(B.1) $$\text{F}3=\frac{\text{n}4-\text{n}3}{\text{rL}1}.$$

Refracting power of the anterior core lens surface

(B.2) $$\text{F}4=\frac{\text{n}5-\text{n}4}{\text{rLC}1}.$$

Reduced interval between the two surfaces

(B.3) $$\text{c}3=\frac{\text{A}4-\text{A}3}{\text{n}4}.$$

Refracting power of the anterior lens system

(B.4) F(3,4)=F3+F4−c3·F3·F4.

(B.2) The Posterior Lens System Refracting power of the posterior core lens surface

(B.5) $$\text{F}5=\frac{\text{n}6-\text{n}5}{\text{rLC}2}.$$

Refracting power of the posterior lens surface

(B.6) $$\text{F}6=\frac{\text{n}7-\text{n}6}{\text{rL}2}.$$

Reduced interval between the two surfaces

(B.7) $$\text{c}5=\frac{\text{A}6-\text{A}5}{\text{n}6}.$$

Refracting power of the posterior lens system

(B.8) F(5,6)=F5+F6−c5·F5·F6.

(B.3) Combining the Anterior and Posterior of the Lens The refracting power of the lens system (FL) is as shown in (2) where

(B.9) $$\text{s}=\frac{\text{H}\left(3,4\right){\text{H}}^{\prime }\left(5,6\right)}{\text{n}5}.$$

### C. The Refractive State of the Eye

The refracting power of the whole eye as (FE) is as shown in (3) where

(C.1) $$\text{cE}=\frac{{\text{H}}^{\prime }\left(1,2\right)\text{H}\left(3,6\right)}{\text{n}3}.$$

The refractive state of the eye (*℞*) is as shown in (4).

*Note*. In the MR image analysis, A7 was measured at the vitreoretinal border; a 130 *μ*m correction for the thickness of the retina was included *ad hoc* to extend the plane of focus approximately to the middle of the photoreceptor layer, as indicated in Table 1 and described by Hughes [64].

### D. Calculating the Principal Points of the Eye

To successfully combine the several optical systems of the eye and account for their spatial relations, it is necessary to derive the principal points (H and H′) of each refractive element: cornea, lens (anterior *and* posterior, if using core model), and their combination.

(D.1) The Cornea System The positions of the principal points A1H(1, 2) and A2H′(1, 2) of the cornea are given by

(D.1) $$\begin{array}{c} \frac{\text{A}1\text{H}\left(1,2\right)}{\text{n}1}=\frac{\text{c}1\cdot \text{F}2}{\text{FC}},\;\;\frac{\text{A}2{\text{H}}^{\prime }\left(1,2\right)}{\text{n}3}=-\frac{\text{c}1\cdot \text{F}1}{\text{FC}} \end{array}$$

and though the first principle point is already referenced to the position of the corneal surface, the second point is referenced to the corneal surface (A1) by

(D.2) A1H'(1,2)=A1A2+A2H'(1,2).

(D.2) The Anterior Lens System The positions of the principal points A3H(3,4) and A4H′(3,4) of the anterior lens surfaces are given by

(D.3) $$\frac{\text{A}3\text{H}\left(3,4\right)}{\text{n}3}=\frac{\text{c}3\cdot \text{F}4}{\text{F}\left(3,4\right)},\;\;\frac{\text{A}4{\text{H}}^{\prime }\left(3,4\right)}{\text{n}5}=-\frac{\text{c}3\cdot \text{F}3}{\text{F}\left(3,4\right)}$$

and when referencing the principal points to the anterior corneal surface (A1),

(D.4) A1H(3,4)=A1A3+A3H(3,4),A1H'(3,4)=A1A4+A4H'(3,4).

(D.3) The Posterior Lens System The positions of the principal points A5H(5,6) and A6H′(5,6) of the posterior lens surfaces are given by

(D.5) $$\frac{\text{A}5\text{H}\left(5,6\right)}{\text{n}5}=\frac{\text{c}5\cdot \text{F}6}{\text{F}\left(5,6\right)},\;\;\frac{\text{A}6{\text{H}}^{\prime }\left(5,6\right)}{\text{n}7}=-\frac{\text{c}5\cdot \text{F}5}{\text{F}\left(5,6\right)}$$

and when referencing the principal points to the anterior corneal surface (A1),

(D.6) A1H(5,6)=A1A5+A5H(5,6),A1H'(5,6)=A1A6+A6H'(5,6).

(D.4) The Anterior and Posterior Lens Systems After combining the anterior and posterior lens systems (when using the core lens model), the positions of the principal points H(3, 4)H(3, 6) and H′(5, 6)H′(3, 6) of the whole lens system are given by

(D.7) $$\begin{array}{c} \frac{\text{H}\left(3,4\right)\text{H}\left(3,6\right)\;}{\text{n}3}=\frac{\text{s}\cdot \text{F}\left(5,6\right)}{\text{FL}},\\ \frac{{\text{H}}^{\prime }\left(5,6\right){\text{H}}^{\prime }\left(3,6\right)}{\text{n}7}=-\frac{\text{s}\cdot \text{F}\left(3,4\right)}{\text{FL}} \end{array}$$

and when referencing the principal points to the anterior corneal surface (A1),

(D.8) A1H(3,6)=A1H(3,4)+H(3,4)H(3,6),A1H'(3,6)=A1H'(5,6)+H'(5,6)H'(3,6).

(D.5) The Whole Eye System Combining the cornea and lens, the positions of the principal points H(1, 2)H and H′(3, 6)H′ of the whole eye system are given by

(D.9) $$\frac{\text{H}\left(1,2\right)\text{H}\;}{\text{n}1}=\frac{\text{cE}\cdot \text{FL}}{\text{FE}},\;\;\frac{{\text{H}}^{\prime }\left(3,6\right){\text{H}}^{\prime }}{\text{n}7}=-\frac{\text{cE}\cdot \text{FC}}{\text{FE}}$$

and when referencing the principal points to the anterior corneal surface (A1),

(D.10) A1H=A1H(1,2)+H(1,2)H,A1H'=A1H'(3,6)+H'(3,6)H'.
